# The progress of assessment methods and treatments of neovascular glaucoma secondary to central retinal vein occlusion

**DOI:** 10.3389/fmed.2023.1280776

**Published:** 2024-01-08

**Authors:** Sheng Qu, Ying Zou, Li Yang, Hong Wu

**Affiliations:** Department of Ophthalmology, The Second Hospital of Jilin University, Changchun, China

**Keywords:** neovascular glaucoma, central retinal vein occlusion, pan-retinal photocoagulation, anti-VEGF, neovascularization

## Abstract

Neovascular glaucoma is a condition that results from central retinal vein occlusion and often leads to blindness. Accurate evaluation and appropriate treatment are crucial for patients. However, there is currently no uniform and clear standard to differentiate between ischemic and non-ischemic central retinal vein occlusion. Also, the assessment of neovascular glaucoma progression is uncertain. Meanwhile, although pan-retinal photocoagulation is a standard treatment to prevent the onset of neovascular glaucoma, its actual efficacy and the timing of intervention remain highly controversial. It is still challenging to balance the risks of side effects in the visual field against the uncertain effectiveness of the treatment. This paper delves into the pathogenesis of neovascular glaucoma to understand the development of therapeutic approaches. By taking into account various assessment criteria of central retinal vein occlusion and neovascular glaucoma over the years, combining functional tests and morphological tests provides the most accurate and rigorous solution. The age of patients, the extent, location, and duration of retinal ischemia are the primary factors that affect the severity and extent of ischemic central retinal vein occlusion and induce serious complications. From the perspective of prevention and treatment, the ischemic index is closely related to the development of neovascularization. The paper provides essential insights into the mechanism, efficacy, complications, and optimal timing of pan-retinal photocoagulation. Comparing the treatment effects of pan-retinal photocoagulation and intravitreal anti-VEGF injections, we suggest a combination of both treatments to explore effective treatment with fewer side effects in the long term. This article details the debate on the above issues and explores ideas for the clinical diagnosis and preventive treatment of neovascular glaucoma that results from ischemic central retinal vein occlusion.

## 1 Introduction

Neovascular glaucoma is the most severe consequence of central retinal vein occlusion (CRVO) that can lead to blindness (1). Therefore, early diagnosis and prompt treatment are essential for patients suffering from neovascular glaucoma (NVG). Further research on the pathogenesis of NVG can aid in the diagnosis and the treatment. Among the various factors contributing to the etiology and pathogenesis of NVG, vascular endothelial growth factor (VEGF) is a significant area of interest. It is responsible for elevated intraocular pressure (IOP), a major symptom of NVG (2).

It is vital to accurately assess patients with CRVO before treatment to reduce the risk of NVG. However, there is still controversy surrounding the criteria for differentiating between ischemic CRVO (iCRVO) and non-ischemic CRVO (3). A unified and effective evaluation standard is expected to be established in the future. During follow-up, it is important to pay attention to the risk factors for the transformation of ischemic to non-ischemic CRVO. Additionally, the severity of CRVO should be evaluated based on high-risk factors for NVG, such as visual acuity and non-perfused areas (4, 5).

The traditional treatment option to prevent ocular neovascularization, especially NVG, is prophylactic pan-retinal photocoagulation (6). However, more research is needed to explore the efficacy and timing of pan-retinal photocoagulation (PRP) (3). It is also important to note that PRP may cause visual field damage to patients with CRVO. The intravitreal anti-VEGF injection has been applied to inhibit neovascular development, but observations have shown that anti-VEGF only delays the development of NVG rather than preventing it (7, 8). Several clinical trials have investigated PRP in combination with anti-VEGF injections, with further research needed in this area (8, 9).

This paper aims to provide an overview of the pathogenesis and diagnosis of NVG. It also offers critical insights into the mechanism, timing, efficacy, and complications of PRP and discusses the treatment effects of PRP and intravitreal anti-VEGF injections. The essay provides ideas for developing clinical diagnosis and treatment of NVG secondary to iCRVO.

## 2 Etiology and pathogenesis of NVG

Tissue hypoxia and pathologic neovascularization of the anterior eye segment cause NVG, resulting in increased intraocular pressure and glaucomatous optic neuropathy (10, 11).

Over the last few decades, the role of VEGF has received extensive attention in RVO. VEGF is a secreted mitogen (12) that promotes angiogenesis and increases vessel permeability (13–16). A recent review (17) concluded that VEGF-VEGFR2 damages tight intercellular junctions by inhibiting occluding and destroys the blood-retinal barrier by activating MMP-9.

In 1995, research using an *in vitro* model showed that VEGF levels from the retina were closely related to the onset of angiogenesis (18). Subsequent studies have demonstrated that when the iris is exposed to VEGF for a sufficient amount of time, non-inflammatory iris neovascularization can occur, potentially leading to NVG in non-human primate eyes (19). Additionally, studies have shown that VEGF-specific antagonists can significantly suppress iris neovascularization in mice and non-human primates with retinal ischemia (20, 21). Pe’er et al. (22) used a VEGF-specific probe to investigate elevated VEGF production in thin whole-eye sections of patients with CRVO, revealing that VEGF is upregulated in response to retinal hypoxia in eyes with CRVO. Furthermore, a study has demonstrated that there is significantly more aqueous VEGF in ischemic CRVO than in non-ischemic CRVO (23), indicating the need for early anti-VEGF therapy in iCRVO patients.

The loss of a significant angiogenesis inhibitor such as pigment epithelium-derived factor (PEDF) can also lead to angiogenesis (24, 25).

As described, we can conclude that the common hypothesis is that a hypoxic environment in ischemia CRVO disrupts the balance between angiogenic stimulating factors (like VEGF) and angiogenic inhibitors (like PEDF) that are closely controlled to maintain homeostasis (25, 26). This disruption can explain the formation of new vessels and fibrous tissue in the root and stroma of the iris (27), leading to the formation of peripheral anterior synechia and secondary angle closure (28). The result is highly elevated intraocular pressure, which can cause glaucomatous optic neuropathy and ultimately lead to NVG (10, 27).

## 3 Risk factors of NVG secondary to RVO

### 3.1 RVO classification

#### 3.1.1 CRVO and BRVO

Retinal vein occlusion (RVO) is classified by the location of the obstruction, primarily involving either the branch retinal vein obstruction (BRVO) or the central retinal vein obstruction (29, 30). Our paper focuses solely on discussing NVG that occurs as a result of CRVO. This is because, BRVO disease rarely results in NVG, unlike CRVO. A prospective observation of 264 eyes with major and macular BRVO over 3–20 years revealed that ocular NV occurred in 28.8% of the major BRVO cases, while none of the macular BRVO cases showed any evidence of ocular NV (31). Firstly, retinal ischemia-perfusion caused by BRVO is not sufficient to cause NV. The severity and extent of retinal ischemia are major factors in ocular NV development in patients with RVO (5, 31). RVO necessitates an ischemic perfused area of over 50% to derive NV (1). BRVO results from venous obstruction of any branch of the central retinal vein; major BRVO is defined as retinal vein obstruction in one quadrant, whereas macular BRVO is defined as obstruction of small intra-macular veins (32). Thus, in patients with BRVO, NV occurs only in severe ischemic major BRVO with extensive capillary non-perfusion (33). Secondly, even if NV is present in branch retinal vein occlusion BRVO, the likelihood of NVG is extremely low. NV in BRVO mostly occurs in the retina and macula (31). But the mechanism of NVG is anterior chamber stenosis caused by NV of the anterior chamber or angle (11).

Further studies may be necessary to develop a consensus standard with both sufficient specificity and sensitivity to differentiate non-ischemic CRVO from ischemic CRVO.

#### 3.1.2 Ischemic and non-Ischemic CRVO

Central retinal vein occlusion (CRVO) is differentiated into ischemic and non-ischemic CRVO by Hayreh (34). It is crucial to differentiate between the two types during the first clinical visit as it helps to improve the effectiveness of treatment and reduce unnecessary damage caused by treatment (3). A prospective clinical study (35) has shown that these two types of CRVO have different clinical features, results, complications, and prognoses, which require different treatments. Previous studies have indicated that the incidence of iris neovascularization (INV), angle neovascularization (ANV), and secondary NVG is significantly higher in the ischemic type than in the non-ischemic type, based on the natural history of the two types of CRVO (4, 31, 36). However, there is still a debate around the criteria for evaluating the methodology and these indicators for assessment.

##### 3.1.2.1 Morphologic tests

As technology has evolved, morphological testing has undergone constant innovation.

In earlier years, fundus fluorescein angiography (FA) was often used as the sole criterion to differentiate between the two types of CRVO to visualize retinal capillary occlusion. In 1993, CVOS (37) identified that the presence of at least 10-disk areas of non-perfusion is a significant risk factor for the development of ischemic or non-ischemic CRVO. Further research conducted in 1997 revealed that visual acuity worse than 20/200, 30 or more-disk areas of obliteration, and moderate to severe venous tortuosity are also crucial factors in predicting the occurrence of CRVO (5). However, data suggests that eyes with less than 30-disk diameters of non-perfusion from fluorescein fundus angiography have a low risk of developing CRVO, provided there are no other risk factors (38). In contrast, eyes with 75 or more-disk diameters of retina capillary occlusion have the highest chance of developing CRVO (38). Therefore, Hayreh (39) argues that the previous standard relying solely on the 10-disk diameters of non-perfusion is not sufficient to accurately distinguish between ischemic and non-ischemic CRVO, which can lead to an imprecise diagnosis, prognosis, and management.

Optical coherence tomography angiography (OCTA) is a fast and trustworthy investigational modality for patients with RVO, which can appraise the deep retina capillary non-perfusion area and morphology of the foveal avascular zone (40–42). OCTA can qualitatively illustrate most of the clinically relevant findings in retinal venous occlusion (40–42).

Central retinal vein occlusion (CRVO) cases with a hyperreflective line located in the outer plexiform layer, called prominent middle limiting membrane sign (p-MLM), had worse final visual outcomes and were more likely to be diagnosed with ischemia CRVO (43).

Even so, within the various limitations of the early stages of CRVO, morphological examinations do not always provide comprehensive and reliable evidence (39).

##### 3.1.2.2 Comprehensive assessment

Hayreh (44) conducted a five-year prospective investigation to establish a standard for distinguishing between ischemic and non-ischemic types. Table 1 shows four functional tests and two morphologic tests required for this purpose (44). The functional tests included visual acuity (VA), visual fields, relative afferent pupillary defect (RAPD), and electroretinography (ERG). The morphologic tests included ophthalmoscopy and FA (44). According to Hayreh (44), by combining all these tests in the early acute stage of almost all cases, we can effectively differentiate ischemic from non-ischemic types.

**TABLE 1:** Functional and morphologic tests for differentiation of CRVO (44).

Test		Parameters
Functional tests	RAPD	> 0.70 log unit
	ERG	b-wave amplitude < 60% of normal or reduced by ≥ 1 SD
	Visual acuity	6/120 or worse
	Visual fields	Central scotoma and peripheral defect or cannot see I2e target of Goldmann perimeter
Morphologic tests	Ophthalmoscopy	≥ Moderate hemorrhages in posterior retina
	Fluorescein fundus angiography	Retinal capillary obliteration

Nonetheless, different researchers have different emphases on classification criteria. Hayreh’s (44) research consistently reported that the combination of RAPD and ERG examination results can increase diagnostic sensitivity and identify 97% of cases. Joussen (45) emphasized the discovery of iris neovascularization or angle neovascularization to diagnose the disease and considered fluorescein angiography the most practical method to detect the degree of ischemia. The Central Vein Occlusion Study Group (CVOS) preferred to rely on the statistical significance of visual acuity rather than initial fluorescein angiography in diagnosis (5).

In conclusion, we still require a significant amount of clinical data to develop more accurate and current diagnostic criteria.

### 3.2 Risk factors for transition to iCRVO

It is worth noting that various studies have indicated that non-ischemic CRVO has the potential to progress into ischemic CRVO at some point (44, 35). Therefore, it would be valuable to conduct research identifying early warning signs of this transition from non-ischemic CRVO to ischemic CRVO.

Age development has been identified as an important factor in the transition of non-ischemic CRVO. Studies show that within 18 months from the first diagnosis of non-ischemic CRVO, individuals aged 65 or older are more likely to convert to iCRVO than other control groups (30).

Meanwhile, the type of CRVO may change with disease progression. Studies have shown that 15% of eyes that were previously perfused turn ischemic within the first four follow-up months (5). A total of 34% of cases become ischemic after three years (5).

The development of ultra-widefield fluorescein angiography (UWFFA) has led to the establishment of the ischemic index (IsI) as a sensitive and specific tool for classifying CRVO types (46, 47). Several studies have demonstrated that a baseline IsI > 35% increases the likelihood of being diagnosed with ischemic CRVO after a systemic examination (46). Additionally, patients with IsI > 35% have a higher probability of converting to ischemic CRVO within one year of onset (46).

Recent research has indicated that the presence of primary open-angle glaucoma in CRVO also significantly indicates an increased risk of developing iCRVO and subsequent NVG, leading to a worse visual outcome (48).

### 3.3 Monitoring of NVG occurrence

After diagnosing iCRVO, it is important to determine the severity of the condition to assess the likelihood of developing NVG. The most significant indicators for this assessment are VA and non-perfusion (5). It is equally important to examine the anterior chamber for NV to prevent the development of NVG. Ultrasound, OCT, and FA can be used for prophylactic examination of NVG.

In 1994, Williamson and Baxter (49) analyzed the relevance between the development of INV and the blood velocities measured by non-invasive color Doppler imaging. This showed that color Doppler imaging could be a routine evaluation for patients examined less than 3 months from the first diagnosis of the occlusion (49).

A retrospective study highlighted that higher central retinal thickness (CRT) on OCT at follow-up is a risk factor for the development of NVG (8). Additionally, the en-face anterior-segment OCTA (AS-OCTA) has been published as a non-invasive method for detecting INV or iris vasculature distribution (50, 51). The data indicates that AS-OCTA quantitative examination corresponds to the results from slit-lamp microscopy and provides more detailed iris vasculature images than iris FA (52, 53).

The extent, location, and duration of retinal ischemia are crucial in determining the degree of perfusion (54). Several studies have reported that OCTA results significantly correlate with non-perfused areas on FA and provide more detailed information about anatomy and blood flow (42, 55, 56). Recently, some analysts have attempted to link the IsI to the degree of non-perfusion to predict the development of NV. A prospective study by Tsui et al. (57) indicated that the IsI calculated from ultra-widefield FA was linked to NV, and eyes with evidence of NV had an IsI over 45%. DeBoer et al. (58) analyzed data from 11 eyes and concluded that patients with low IsI values in the peripheral areas were less likely to develop NVG, with no significant increase in non-perfused areas. One study by Nicholson et al. (4) also reported that posterior pole non-perfused areas of more than 10 indicate a greater risk of neovascularization compared with peripheral non-perfused areas greater than 10. Wykoff et al. (59) pointed out that eyes with anterior NV showed a weaker correlation with the level of retinal non-perfusion than posterior segment NV. It would be helpful to have more information on the connection between IsI and neovascularization development to establish a greater degree of accuracy on whether IsI can be applied in the prediction of NVG.

Currently, there is still no reasonable standard that can be proven by clinical practice, which requires a lot of clinical trials to verify.

## 4 PRP in iCRVO

### 4.1 PRP effect in reducing INV/ANV

There is a limited number of historical studies on the effect of a single PRP in the development of INV/ANV. By analyzing several highly credible prospective studies conducted in the 1990s, we can see contrasting perspectives.

One survey by the CVOS group N found that patients did not see a significant benefit from PRP before the occurrence of INV/ANV (38). Instead, they found that laser treatment was effective in reducing anterior segment neovascularization when INV/ANV appeared during strict and timely follow-up (38).

A study conducted by Hayreh et al. (60), over a long period of time showed that PRP did not have any positive effect in preventing NVG as a result of iCRVO. On the contrary, it led to a certain degree of peripheral visual field degradation in many treated eyes (60). The study indicated that the incidence of INV was significantly lower in the laser group than in the non-laser group, but only when PRP was given within three months after the onset of iCRVO (60). However, their report showed no statistically significant difference in the incidence of NVG between the laser and non-laser groups (60). This result may be explained from three aspects. Firstly, according to the pathology of NVG, the obstruction of trabecular meshwork by fibrovascular tissue proliferating onto the anterior chamber angle plays an equally significant role (61), usually secondary to widespread posterior segment ischemia (62). Hence, both INV and ANV play a critical role in developing NVG in most cases (38). However, the studies by Hayreh et al. (60) denied the effect of early PRP on ANV, which may affect the positive impact of PRP on NVG. Secondly, the lower possibility of NVG in iCRVO may decrease the efficacy of PRP statistically. Finally, the study by Hayreh et al. (60) showed that PRP may have missed the best time for treatment after INV occurred due to the absence of close follow-up. When high IOP has formed, it is difficult for PRP to have an expected therapeutic effect on NVG (3, 60).

Some researchers also questioned the necessity of PRP in iCRVO, considering the development of anterior segment NV and the result of NVG. In 2012, Hayreh and Zimmerman (63) published a strict paper defining iCRVO by the criteria from their previous study. The data showed that within 6 months of the onset of ischemic CRVO, the incidence rate of iris NV was 49%, angular NV was 39%, and NVG was 29% (63). Despite different study designs and the vague standard of iCRVO, we can still conclude from most studies (31, 64–66) that no more than 49% of iCRVO cases will result in INV or/and ANV, and not all INV or/and ANV will lead to the emergence of NVG. The data suggests that about 55% of patients with ischemic CRVO may never develop an NVG outcome (1).

### 4.2 Side effects of PRP

Excessive PRP spot density may lead to a reduction or defect in the peripheral field of view.

In 1990, Hayreh et al. (60) presented an analysis and discussion on how PRP produces apparent constriction and a noticeable peripheral visual field loss. This result led to a debate on whether PRP does more harm than good to most eyes with iCRVO. Oosterhuis and Sedney (67) and Laatikainen et al. (68) also found severe visual field constriction in the treated eyes in their investigations into PRP treatment in CRVO.

Visual fields will also decline in the natural history of CRVO. A study by Hayreh et al. (69) found that 55% of patients with ischemic CRVO have a 55% probability of developing scotoma within 3 months of onset, with most being central blind spots. Besides, 17% of patients with ischemic CRVO present with peripheral visual field defects at first diagnosis (69). Another study by Hayreh (70) reported that in patients with CRVO and moderate to severe visual field defects at the beginning, the occurrence of NVG deteriorated the visual field of 76% of the eyes (70). In contrast, 38% of the eyes without NVG had visual field deterioration.

As Hayreh et al. (60) mentioned, if patients with iCRVO who will not develop into NVG receive unnecessary PRP treatment, the loss of the peripheral visual field caused by PRP treatment will worsen the vision of patients with central scotoma shown by CRVO. This proves that PRP has no value in treatment and is seriously detrimental.

It’s important to note that although the occurrence of NVG is not very common and PRP has the potential to cause visual field and vision problems, NVG is a serious and irreversible condition that can lead to blindness. Therefore, it’s not advisable to avoid PRP prophylaxis simply out of caution. These findings also have significant implications for understanding the necessity of follow-up to determine the extent and severity of iCRVO to decide when PRP is suitable for the case.

### 4.3 Best time for PRP

As mentioned earlier, the findings of CVOS research support the idea that prophylactic PRP may not entirely prevent INV/ANV (38). But PRP is still a recommended option when INV/ANV occurs to prevent NVG (38). Therefore, guidelines by the European Society of Retina Specialists suggest that “PRP should be recommended only after iris neovascularization becomes visible, requiring weekly or biweekly follow-up of patients with extensive capillary non-perfusion.” (6) However, since it is often difficult to maintain close follow-up, guidelines suggest early prophylactic PRP (within 90 days of the onset of the CRVO) as a second choice to take precautions against INV in ischemic CRVO (6), as confirmed by the results of the study by Hayreh et al. (60).

**FIGURE 1:**
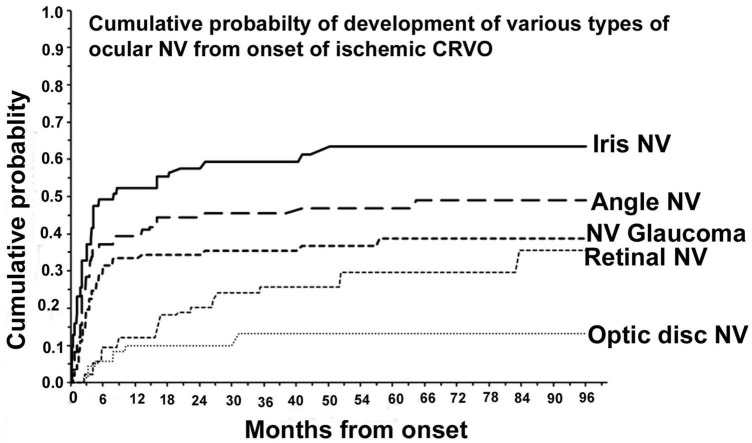
Cumulative probability of various types of ocular NV after diagnosing ischemic CRVO. Reprinted with permission from Hayreh (3). Copyright© 2021 Elsevier Ltd.

Observing the eyes with iCRVO closely for the first 6 months is crucial to predict early NVG (63). After the first 6 months, observation can be less frequent (63). Studies conducted by Hayreh (29) in 1983 and by Zimmerman (63) in 2012 indicate that anterior segment NV is most likely to occur during the first 6 to 7 months, as shown in Figure 1. After this period, the chances of its occurrence are minimal (29, 63). Additionally, over 80% of cases of NVG develop within 6–8 months (29, 63).

According to Hayreh et al.’s (29) research, early prophylactic PRP within 90 days is as effective as PRP after INV appears, since the incidence of INV increases rapidly in the first 3 months. Researchers have found that NVG, also known as 100-day glaucoma, usually occurs within three months of iCRVO onset by analyzing the cumulative chance of NVG secondary to iCRVO (1, 29, 69).

### 4.4 Mechanism of PRP

Neovascular glaucoma is a severe and blinding disease that can result from ischemic CRVO, which occurs when the trabecular meshwork becomes blocked by proliferated fibrovascular tissue (71). INV and ANV are both indicators of non-perfusion and can increase the risk of developing NVG (60, 72). For many years, researchers have recommended PRP as a preventive measure against INV/ANV in patients with iCRVO (3, 6). Specifically, they have suggested PRP as a standard treatment after iCRVO to prevent the onset of NVG (3, 6). However, it is still debatable whether it is wise for patients with iCRVO to receive PRP to reduce the risk of NVG.

The treatment mechanism of photocoagulation has two main assumptions: (1) to establish a hyperoxic environment causing vasoconstriction (73, 74), and (2) to adjust the angiogenic stimulating factors and angiogenic inhibiting factors to suppress neovascularization.

Oxygen pressure in the retina is determined by the balance between oxygen diffusion by retinal arterioles and oxygen consumption by retinal tissue (75). Photocoagulation damages the pigment epithelium-photoreceptor complex (73), which consumes oxygen. Simultaneously, it destroys viable retinal tissue to increase oxygen diffusion from the choroid (76). Animals breathing air or 100% O_2_ at atmospheric pressure show that the photocoagulated retinal areas have a higher partial pressure of oxygen than the normal retina (77–80). Therefore, photocoagulation creates a hyperoxic state that causes retinal vasoconstriction (73, 74).

Retinal photocoagulation affects VEGF and PEDF levels. With the in-depth discovery of VEGF, it has been found that the retinal pigmented epithelium (RPE), astrocytes, Müller cells, vascular endothelium, and ganglion cells create this growth factor (81). PRP can destroy many retinal cells to restrain NV and the NVG. Additionally, a study has demonstrated a temporal alteration and transcriptional activity caused by retinal laser photocoagulation in the area of VEGF production (82). However, Itaya et al. (83) showed the contrary result: the growth factor, mainly from the recruited monocytes, is upregulated at the early stage in the sensory retina and RPE-choroid after retinal scatter laser photocoagulation. Nevertheless, upregulation of angiogenic inhibitors, like PEDF and TGF-beta 2, has been found following PRP (84–86). Further experimental investigations are necessary to estimate whether PRP downgrades VEGF and upgrades PEDF levels.

When considering the use of PRP as a preventive treatment for NVG, there are two aspects to consider. Firstly, it is still controversial whether PRP is a suitable option for reducing the growth of anterior segment NV (1, 3). Secondly, we need to consider the impact of laser treatment on vision (3, 60, 87). By taking both of these points into account, we can evaluate the pros and cons of using PRP in iCRVO.

## 5 PRP and anti-VEGF

### 5.1 Anti-VEGF is not a substitute for PRP

To date, several studies have increasingly suggested anti-VEGF injections to abate INV and thus make it play a role in the prevention and treatment of NVG (88). Several researchers have reported the regression of iris neovascularization with intravitreal and intracameral bevacizumab (89–91). Likewise, some data have investigated that single anti-VEGF therapy plays a significant role in reducing the increase of IOP (92–94). These pieces of evidence suggest that the aggressive blockade of VEGF may be effectively applied to prevent NVG in iCRVO. However, it is still controversial whether anti-VEGF can completely replace traditional PRP therapy.

Blocking vascular endothelial growth factor does not stably prevent neovascular complications, but only delays their onset (7, 8). A small-scale study by Inatani et al. (94, 95) concluded that intravitreal aflibercept injection leads to meaningful IOP reductions in 5 weeks. But another study found that high levels of CRT remained at 1-month follow-up after initial IVB therapy, demonstrating the poor efficacy of single anti-VEGF therapy alone (8). A long-term comparison study also pointed out that the decrease of IOP has no difference between the eyes with and without anti-VEGF in the longer follow-up (96). Therefore, in a randomized controlled study of ranibizumab application in CRVO, Campochiaro et al. (97, 98) reported that monthly ranibizumab injections could reduce the proportion of eyes with retinal capillary obstruction and the progression of retinal non-perfusion. However, the proportion of eyes with retinal non-perfusion increased after six months when researchers changed monthly ranibizumab injections to pro re nata (PRN) injections (97). Sophie (99) described the same result: resumption of the PRN blockage of VEGF would reverse retina non-perfusion. NVG statistically occurs an average of 7 months after the last anti-VEGF injection (8, 100). Namely, intravitreal anti-VEGF functionally delays the occurrence time of the neovascularization compared to the natural history in patients with iCRVO (100, 101).

The short-term and incomplete inhibitory effect of anti-VEGF can be attributed to the short half-life (102, 103), the complexity of multiple pathways in ocular angiogenesis (26), and the limitation that anti-VEGF only works on the existing VEGF (104).

As can be seen, VEGF inhibition cannot stably control the development of inner retinal non-perfusion and IOP for a long time. Comparing anti-VEGF therapy with PRP to without PRP, researchers illustrated that PRP substantially plays a leading role in delaying and decreasing the need for IOP control instead of bevacizumab administration (96). All of the above suggests anti-VEGF as adjuvant therapy instead of an alternative treatment for PRP.

### 5.2 PRP combined with anti-VEGF

Clinical experts recommend anti-VEGF drug injections taken within 5–14 days before undergoing vitreous surgeries (105). To begin with, the characteristic of anti-VEGF to rapidly regressing NV prolongs the PRP treatment time window. PRP usually takes several weeks to make efforts (106). While anti-VEGF treatment can effectively bring the IOP under control within 1 month (107). For another, injecting anti-VEGF before PRP treatment reduces postoperative complications, including hyphema and NVG (93, 108–110). High levels of VEGF in the perioperative period predict postoperative vitreous hemorrhage and neovascular glaucoma (111, 112). In order to prevent recurrence of NV-related vitreous hemorrhage (VN) after PRP, intravitreal injection of anti-VEGF every 3–4 months is recommended (113).

Nevertheless, it is unclear whether combination therapy can reduce PRP side effects and improve efficacy. Several studies have achieved effective long-term treatment by combining known endogenous angiogenesis inhibitors with conventional PRP therapy (9, 114, 115). Further, the treatment program’s effectiveness and potential adverse effects were evaluated through tests such as assessment of VA, control of IOP, and evaluation of retinal function. Unexpectedly, PRP with anti-VEGF treatment does not improve or deteriorate the low VA outcomes significantly (9, 96, 116). Regarding the improvement of IOP control, researchers hold different views. Combination therapy has several benefits for treating IOP conditions. Firstly, it results in a quicker and more consistent reduction in IOP and a faster regression of NV (9). Additionally, studies conducted by Vasudev et al. (114) have shown that combination therapy helps maintain open-angle documented by fundoscopy over time. However, it is worth noting that combination therapy does not significantly improve IOP reduction in the long term (96, 116). Evaluating the retinal function by full-field ERG, the outcomes demonstrated that the photoreceptor function in patients with NVG was decreased by bevacizumab therapy (116), which leads to worse side effects of therapy. As described, while anti-VEGF creates conditions for PRP, further studies are needed to confirm the gainful effects of combination therapy in large samples of long-term research.

In recent years, intracameral injections of anti-VEGF have been proposed to achieve quicker and more precise therapeutic outcomes (117). VEGF primarily derives from the retina, with possible supplementary sources in the ciliary epithelium (118). In primate eyes, intravitreal anti-VEGF injections resulted in the highest drug concentrations in the iris and atrial angle on day 1, and in the ciliary body on day 4 (119). Administration of intracameral anti-VEGF injections seems to have more targeted therapeutic effects for INV and ANV. Further trials are needed to confirm the benefits of this surgical approach. Meanwhile, when injecting drugs into silicone oil-filled eyes, it is tough to ensure proper concentration in the vitreous cavity (117). Therefore, injecting drugs into the anterior chamber is a viable option for treating NVG in these patients (117).

## 6 NVG treatment

Glaucoma is classified into three stages according to the progression of the disease (109). Pre-glaucoma (Phase I) is characterized by the presence of INV with normal IOP levels. With elevated IOP, the NVG is divided into open-angle (Phase II) and closed-angle (Phase III) types.

The primary objective of NVG therapy is to inhibit NV and decrease IOP. If PRP and anti-VEGF treatments are ineffective at Phase I, conventional glaucoma medications and surgical procedures are suggested.

Medications for glaucoma are divided into two main categories: IOP-lowering and anti-inflammatory. Drugs that topically reduce aqueous production include β adrenoceptor blockers, α-2 adrenoceptor agonists, and carbonic anhydrase inhibitors (109). If the target IOP is still not reached with medication, there are surgical options to help with the obstruction of aqueous humor outflow. These options include trabeculectomy, glaucoma drainage devices, and minimally invasive internal drainage procedures (120). In cases where the first surgery fails, refractory glaucoma can be treated with surgical options that reduce the amount of aqueous humor produced by the ciliary, such as cyclophotocoagulation or cyclocryotherapy (120).

## 7 Conclusion

In the medical field, PRP remains a commonly used treatment for NVG prophylaxis. It is recommended that patients undergo PRP as soon as possible after being diagnosed with iCRVO, ideally within 90 days. However, the effectiveness and safety of PRP in treating and managing NVG secondary to iCRVO is still uncertain, and more randomized controlled clinical trials are needed to determine the optimal treatment time and reliable indications for PRP. Additionally, PRP can cause side effects and complications, such as losing the peripheral visual fields (60). Therefore, further research is needed to establish definitive evidence of the etiopathogenesis of NVG and the mechanism of PRP treatment. This will help promote the development of PRP or other advanced treatment options.

Intravitreal anti-VEGF injections are performed before vitrectomy to rapidly ablate neovascularization and to allow for PRP treatment while reducing the risk of surgical complications such as vitreous hemorrhage (105). In addition to the combination of PRP and known anti-VEGF treatments, further research could be done to assess the effectiveness of new substances that regulate angiogenesis, such as PEDF (24, 25), and inflammatory proteins, such as interleukin-6 (121, 122). Recently, a new emulsion-formulated antisense oligonucleotide has been developed to prevent NV by blocking insulin receptor substrate-1 (IRS-1) in the intraocular retina with topical eye drops (123). This treatment, which has attracted significant attention in preventing NVG, is currently in phase II/III trials and shows excellent potential (123). Gene therapy for endogenous angiogenic inhibitors also has attractive prospects, such as plasminogen kringle 5 (124, 125), recombinant adeno-associated virus (126), gene transfer of prolyl hydroxylase domain 2 (127), and more.

It is important to note that there are several limitations in clinical diagnosis and follow-up due to the subjective consciousness of patients and hospital equipment. Patients often do not seek preventive treatment, and even fewer complete follow-up procedures on time. To assess ischemic and non-ischemic CRVO, visual acuity and RAPD examinations are commonly conducted. However, inadequate inspection can lead to incorrect assessments and limited treatment options. Additionally, insufficient attention to angle NV can delay the preventive treatment of NVG. A study has shown that ANV can occur without pupillary margin involvement in CRVO, indicating the need for screening gonioscopy (65).

In conclusion, the effectiveness of PRP in preventing NVG as a complication of CRVO still requires further exploration. A substantial amount of evidence is needed, ranging from basic research to clinical practice. Diagnostic methods and prophylactic protocols for NVG also need to be further improved.

## Author contributions

SQ: Writing – review & editing, Conceptualization, Investigation, Writing – original draft. YZ: Investigation, Writing – review & editing. LY: Writing – review & editing. HW: Conceptualization, Funding acquisition, Methodology, Project administration, Writing – review & editing.
